# Modified Poly(Heptazine Imides): Minimizing H_2_O_2_ Decomposition to Maximize Oxygen Reduction

**DOI:** 10.1021/acsami.2c14872

**Published:** 2022-10-31

**Authors:** Andrea Rogolino, Ingrid F. Silva, Nadezda V. Tarakina, Marcos A. R. da Silva, Guilherme F. S. R. Rocha, Markus Antonietti, Ivo F. Teixeira

**Affiliations:** † Department of Chemistry, Federal University of São Carlos, Washington Luis Highway, s/n Km 235, São Carlos13565-905, São Paulo, Brazil; ‡ Galilean School of Higher Education, University of Padova, Via Venezia 20, Padova35131, Italy; § Department of Colloid Chemistry, Max Planck Institute of Colloids and Interfaces, Am Mühlenberg 1, Potsdam14476, Germany

**Keywords:** photocatalysis, carbon nitrides, hydrogen peroxide, poly(heptazine imides), oxygen reduction

## Abstract

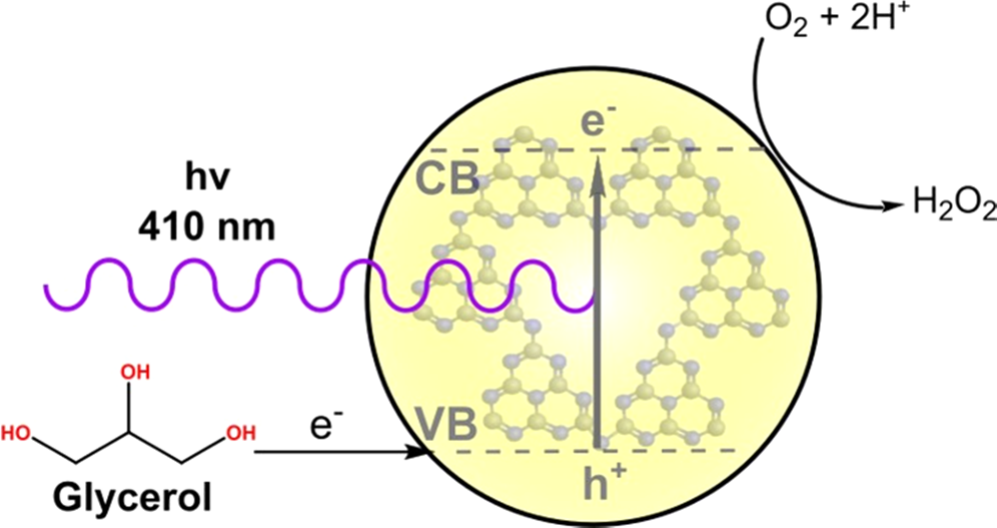

Photocatalysis provides a sustainable pathway to produce the consumer chemical H_2_O_2_ from atmospheric O_2_ via an oxygen reduction reaction (ORR). Such an alternative is attractive to replace the cumbersome traditional anthraquinone method for H_2_O_2_ synthesis on a large scale. Carbon nitrides have shown very interesting results as heterogeneous photocatalysts in ORR because their covalent two-dimensional (2D) structure is believed to increase selectivity toward the two-electron process. However, an efficient and scalable application of carbon nitrides for this reaction is far from being achieved. Poly(heptazine imides) (PHIs) are a more powerful subgroup of carbon nitrides whose structure provides high crystallinity and a scaffold to host transition-metal single atoms. Herein, we show that PHIs functionalized with sodium and the recently reported fully protonated PHI exhibit high activity in two-electron ORR under visible light. The latter converted O_2_ to up to 1556 mmol L^–1^ h^–1^ g^–1^ H_2_O_2_ under 410 nm irradiation using inexpensive but otherwise chemically demanding glycerin as a sacrificial electron donor. We also prove that functionalization with transition metals is not beneficial for H_2_O_2_ synthesis, as the metal also catalyzes its decomposition. Transient photoluminescence spectroscopy suggests that H-PHIs exhibit higher activity due to their longer excited-state lifetime. Overall, this work highlights the high photocatalytic activity of the rarely examined fully protonated PHI and represents a step forward in the application of inexpensive covalent materials for photocatalytic H_2_O_2_ synthesis.

## Introduction

Hydrogen peroxide (H_2_O_2_) is a simple inorganic chemical whose properties make it extremely versatile and valuable for not only consumer applications but also industrial processes. It is extensively used in the paper industry as a bleaching agent, and its well-known bactericidal and virucidal activity is relevant to water treatment as well as healthcare.^1−3^ Moreover, in the past few years, the market of H_2_O_2_ has witnessed a dramatic increase because of a surged interest in its potential for fuel cells, where one single substrate would generate anodic (H_2_O_2_ → O_2_ + 2e^–^ + 2H^+^) and cathodic (H_2_O_2_ + 2e^–^ + 2H^+^ → 2H_2_O) current.^4^ Finally, replacing conventional oxidants in organic chemistry with hydrogen peroxide is highly desirable to fulfill green chemistry goals, as H_2_O_2_ is relatively safe, easy to store, and nonpolluting, since it generates water as the only byproduct.^5^ In 2020, the hydrogen peroxide market size was $1.64 billion and is forecast to increase to $2.2 billion in 2028.^6^ Hydrogen peroxide is currently produced in over 3.5 million metric tons per year through the so-called anthraquinone process. In this method, anthraquinone, dissolved in a proper organic solvent, is first reduced to anthrahydroquinone by bubbling gaseous H_2_ over a Pd catalyst. Anthrahydroquinone is then the reducing agent in H_2_O_2_ production from streamed O_2_. This step occurs at 30–60 °C under mild pressure. Anthraquinone is formed back to close the cycle, while H_2_O_2_ is subsequently isolated in water by solvent extraction.^7^ Although the industrial process yields H_2_O_2_ in good amounts, it has several drawbacks in terms of sustainability: in particular, the use of (i) pressurized and flammable H_2_, (ii) noble metal catalysts, and (iii) large amount of organic solvents to be processed by liquid–liquid extraction. Consequently, in recent years, much effort has been put to develop efficient catalytic methods of H_2_O_2_ production with high atom efficiency, that is, involving the conversion of only hydrogen- and oxygen-containing molecules, limited waste, and clean catalysis.^8−10^ Both electrocatalytic and photocatalytic approaches have been investigated to allow innovative routes to H_2_O_2_ synthesis, which can be summarized in three categories: (i) 2e^–^ oxygen reduction reaction (ORR), (ii) 2e^–^ water oxidation reaction (WOR), and (iii) direct synthesis by H_2_ and O_2_ comproportionation.^11^ The redox couples most relevant to these reactions are reported below (eqs 1–5). If not stated differently, all of the reduction potentials in this document are given relative to the standard hydrogen electrode (SHE).
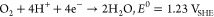
1
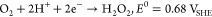
2
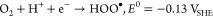
3
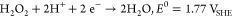
4
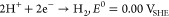
5The 2e^–^ WOR route has been largely overlooked, likely because of the high reduction potential of the back reaction (eq 4), which makes the reaction thermodynamically unfavorable. Nevertheless, a few photoelectrocatalytic examples have been reported.^12,13^ However, most of the research in H_2_O_2_ synthesis so far has focused on ORR (eq 2). Light-assisted conversion of abundant oxygen into a value-added molecule is a promising strategy to convert and store solar energy into chemical energy.^14^ Graphitic carbon nitrides are robust and easily synthesized materials containing conjugately bonded carbon and nitrogen atoms. Antonietti et al. paved the way for heterogeneous photocatalytic applications of metal-free carbon nitrides when they first reported its activity in visible light-assisted hydrogen generation from water.^15^ Carbon nitrides exhibit excellent features for sustainable heterogeneous photocatalysis: (i) cheap and straightforward synthetic procedures, (ii) metal-free, organocatalytic conditions, (iii) high thermal stability, (iv) tunable morphology, (v) large band gap and tunable visible light absorption.^16,17^ Recently, carbon nitrides turned out to be interesting materials for H_2_O_2_ in synthesis in terms of enhanced selectivity. The triazine and heptazine units in carbon nitrides are believed to kinetically enhance the selectivity through the formation of 1,4-endoperoxide species, thus leading to a suppression of HOO^•^ production by 1e^–^ ORR (eq 3) in favor of 2e^–^ ORR (eq 2). Hirai et al. first demonstrated this by means of Raman spectroscopy.^18^

Poly(heptazine imides) (PHIs) are improved, highly crystalline carbon nitrides, which recently gained popularity as suitable scaffolds to host isolated metal cations.^19,20^ Here, we show that the sodium-functionalized PHI (Na-PHI), obtained via a straightforward synthesis, and the fully protonated PHI (H-PHI) exhibit very high activity for photocatalytic ORR to H_2_O_2_ under visible light and mild conditions. Conversely, we prove that PHIs functionalized with transition metals are detrimental to H_2_O_2_ production because they are likely to favor its further disproportionation to H_2_O and O_2_. Finally, measurements of transient photoluminescence suggest that the enhanced performance of H-PHIs might be attributed to a lower extent of charge recombination.

## Results and Discussion

### Synthesis of Metal-Doped Poly(Heptazine Imides) and Characterization

Metal-doped poly(heptazine imides) (M-PHIs) were synthesized from a common precursor where sodium is the counterion (Na-PHI). This is readily synthesized via a straightforward reported protocol: sodium chloride and melamine are mixed in 10:1 proportion, ground, and calcined under an inert gas flux.^21^ It should be noted that unlike examples of carbon nitrides doping aimed at changing the electronic structure,^22^ Na ions in PHIs have mainly a templating role. Sodium cations intercalate between the planes of the polymeric structure and are coordinated by electrostatic interactions with deprotonated nitrogens (Figure S1). M-PHIs are easily prepared by taking advantage of this labile coordination.^23,24^ Such ions exhibit sufficient mobility to be washed with an electrolyte solution and replaced by harder cations of sufficiently small radius to fit into the graphitic scaffold. Divalent and trivalent cations are hard enough to coordinate more tightly to the anionic holes. Here, earth-abundant Fe(III), Ni(II), and Co(II) and noble Ru(III) were investigated. Metals were directly incorporated from aqueous solution of their chlorides. Molar concentrations of the solutions were low enough to guarantee a 1.5% or lower metal loading in the final material. Samples of Fe-PHIs with different metal loadings (1, 0.5, 0.1, and 0.02%) were synthesized to investigate the effect of the metal loading on the catalysis. All characterizations were referred to Fe-PHI-1%. Dried Na-PHIs were mixed with these solutions and subsequently centrifuged and washed. The effective metal loadings achieved were measured by ICP-OES and are listed in Table S1. Together with metal-doped PHIs, the fully protonated poly(heptazine imides) (H-PHIs) were investigated. Protonation of nitrogens prevents cations from undergoing coordination, and the resulting carbon nitride can be formally thought as a PHI where metal cations are replaced by protons. H-PHIs are obtained when concentrated hydrochloric acid is used instead of a metal chloride solution in the cation-exchange protocol.

**Figure 1:**
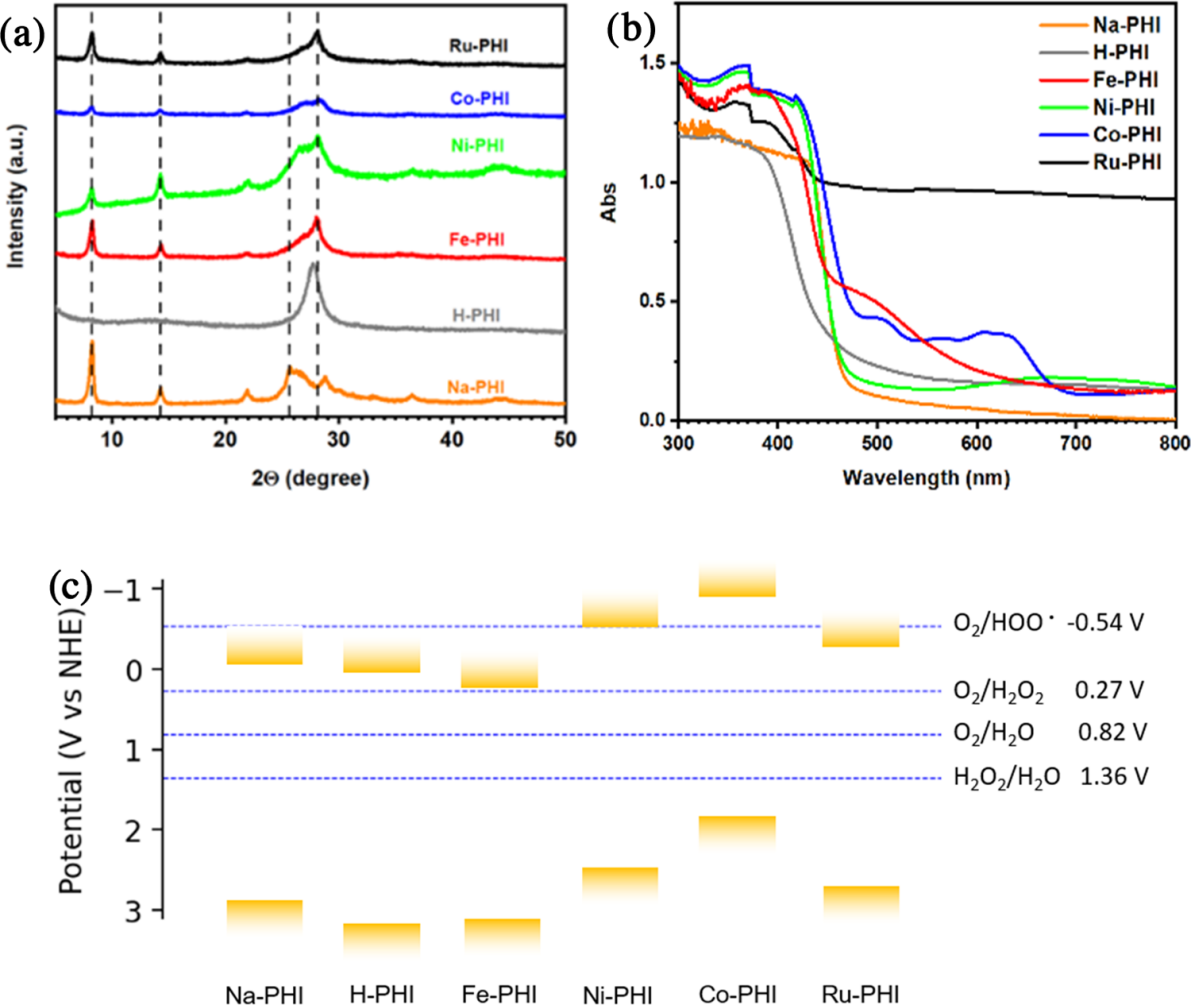
(a) PXRD diffractograms of the prepared poly(heptazine imides). (b) Diffuse reflectance UV–vis spectra of the prepared poly(heptazine imides). (c) Position of the conduction and valence bands of the prepared poly(heptazine imides) at pH = 7. Standard redox potentials at pH = 7 of the most relevant redox couples are reported on the right.

Prepared materials were first characterized by PXRD to investigate the influence of the synthetic protocols on crystallinity (Figure 1a). Reflections at 8.2° (*d* = 10.8 Å) and 14.3° (*d* = 6.2 Å) correspond to the (100) and the (110) planes of the trigonal lattice, respectively, indicating formation of the poly(heptazine imide) structure.^19,21^ These reflections were retained in all materials except H-PHIs, pointing toward the loss of in-plane order during the treatment with concentrated hydrochloric acid. The region between 25 and 29° in the pattern of Na-PHIs consists of several overlapping peaks with the peak at about 27° (*d* = 3.38 Å) corresponding to interlayer distance in poly(heptazine imides); one can clearly see a shift of this peak during an ion exchange reaction with metal chloride solutions, suggesting that cation replacement highly affects the spacing between adjacent layers along *c*-direction in the structure. The transmission electron microscopy study of Na-PHIs and H-PHIs confirms a high degree of crystallinity of Na-PHIs (Figure S2).

Metal-containing carbon nitrides appeared as colored powders. Na-PHIs, Ni-PHIs, and Co-PHIs retained the typical yellow color of graphitic carbon nitrides, Fe-PHIs turned into a dark yellow-orange, while Ru-PHIs were black. When Na-PHIs were mixed with acid to make H-PHIs, they immediately became white. Color differences were also reflected in the diffuse reflectance UV–vis (DR-UV–vis) spectra collected (Figures 1b and S2b). Spectra were elaborated in the framework of Kubelka–Munk theory to extrapolate energy band gaps. Tauc plots of Na-PHIs and H-PHIs are provided in Figure S3b. The CB edge potentials were extrapolated from Mott–Schottky plots (Figure S3c shows Mott–Schottky plots of Na-PHIs and H-PHIs). Table S2 and Figure 1c summarize the results from the combined Tauc and Mott–Schottky analysis. All synthesized PHIs have an in principle suitable CB edge potential to drive two-electron ORR. More interestingly, conduction bands for all catalysts, with the exception of Co-PHIs, lie at more positive potentials than the O_2_/HOO· reduction potential. As a consequence, 1e^–^ ORR is suppressed in favor of 2e^–^ ORR to H_2_O_2_. Although conduction bands lie well above O_2_/H_2_O and H_2_O_2_/H_2_O potentials, these reactions might have slower kinetics. Indeed, it should be reminded that no conclusions can be drawn on the efficiency of catalysis from pure thermodynamic considerations.

**Figure 2:**
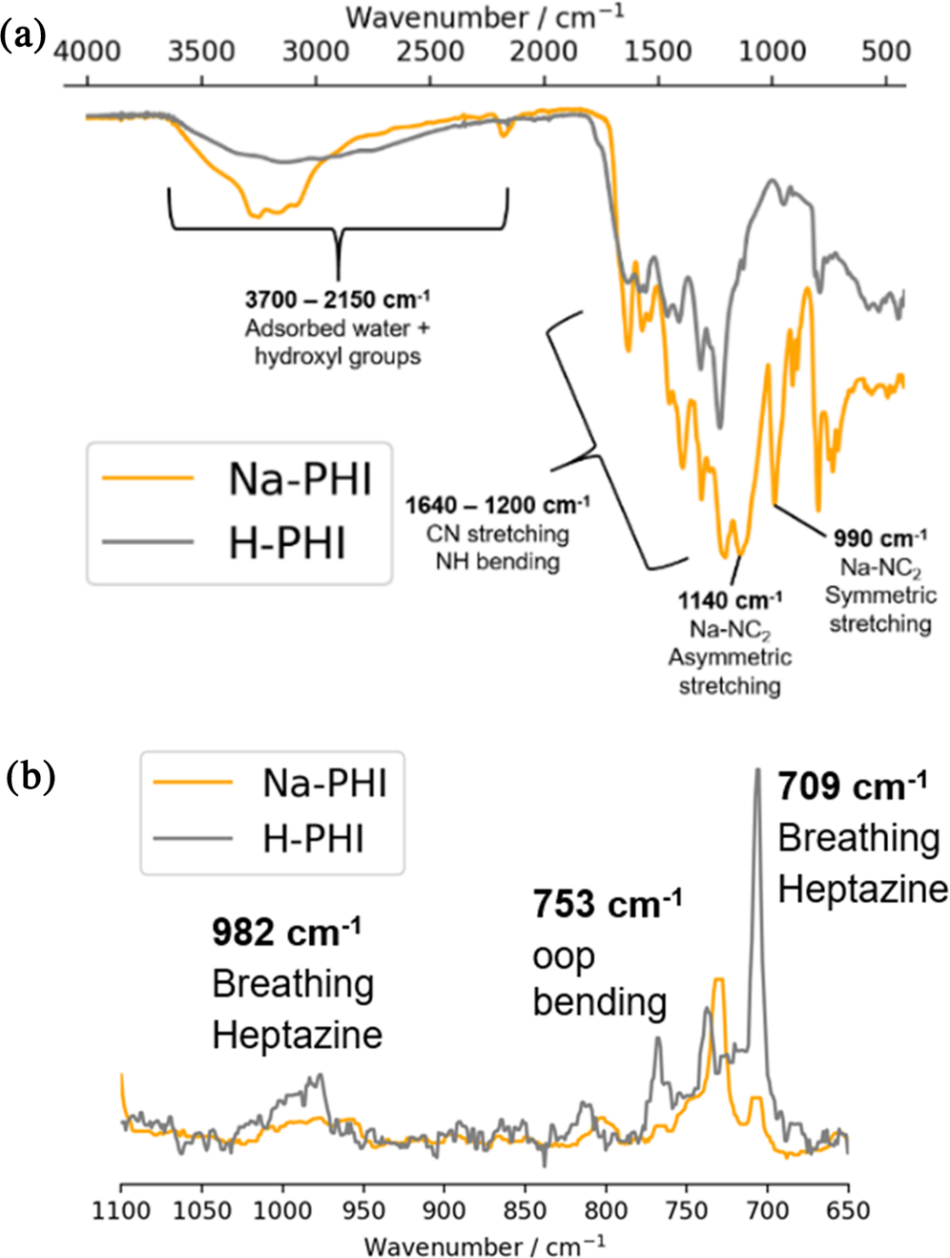
(a) FTIR and (b) Raman spectra of Na-PHIs and H-PHIs.

The precursor Na-PHI and the fully protonated H-PHI were subjected to further characterization. The FTIR spectrum of Na-PHIs is in very good agreement with those reported in the literature (Figure 2a).^21^ In particular, the region between 1200 and 1640 cm^–1^ comprising multiple C–N stretching and N–H bending vibration modes is well defined. Both Na-PHIs and H-PHIs present a broad band between 2150 and 3700 cm^–1^ due to adsorbed water molecules. Na-PHIs are characterized by two sharp peaks at 1140 and 990 cm^–1^, which were attributed to the asymmetric and symmetric stretching of Na–NC_2_ bonds in the metal-heptazine unit. Interestingly, these peaks disappear in H-PHIs, confirming the successful removal of coordinated sodium ions due to the total protonation of nitrogens. Raman spectroscopy identified fingerprint IR-inactive vibration modes, especially the breathing of heptazine units at 709 and 982 cm^–1^, together with a peak at 753 cm^–1^, which was assigned to an out-of-plane bending of the graphitic domains (Figure 2b).^18^ Deconvoluted XPS spectra of Na-PHIs and H-PHIs reproduced the results from previous reports (Figure S4).^21^

### Catalytic Screening and Optimization

Photocatalytic ORR was assessed in a pure oxygen/water mixture. An LED lamp with emission at 410 nm was chosen to irradiate close to the maximum absorbance of all poly(heptazine imides). Glycerin was selected as an electron donor, which is a sustainable but complicated agent not activated by graphitic carbon nitride for instance. It was however successfully applied in a previous work involving single-atom-doped carbon nitrides.^25^ Glycerin is a desirable alternative to the standard trialkyl amine SEDs because its synthesis as a byproduct of the biofuel industry is forecasted to increase in the future. Glycerin undergoes 2e^–^ oxidation to dihydroxyacetone or glyceraldehyde while transferring electrons to the photocatalyst. Catalytic conditions were first optimized using Fe-PHI-0.1% as a reference (Table 1). Evaluated parameters were the method of oxygen supply, the reaction volume, the sacrificial electron donor, and the pH given by the addition of different acids and bases. To find out whether oxygen was consumed too rapidly to equilibrate between the gas and the liquid phase, an oxygen reservoir in a balloon was placed above the vial. The presence of a constant oxygen supply, however, made no significant difference (entry 2). Glycerin and methanol gave comparable yields when used in the same w/w concentration as sacrificial electron donors, while only a negligible amount of H_2_O_2_ was detected without any SED (entries 5 and 6). Most interesting was the effect of pH modulation. Oxygen reduction to hydrogen peroxide is expected to be favored in acidic media (O_2_ + 2e^–^ + 2H^+^ → H_2_O_2_). However, acid catalysis might increase the H_2_O_2_ decomposition rate as well.^26,27^ The addition of different acids and bases in comparable molar amounts had no positive effect on H_2_O_2_ yield (entries 7–11). The reaction proceeded well without further additives in a neutral pH. The addition of a buffer was not helpful either. When the glycerin was diluted in a buffered NaH_2_PO_4_/Na_2_HPO_4_ solution, no H_2_O_2_ was detected at all. This is likely due to a salting out effect, where glycerin solubility in water decreases due to increased ionic strength. The pH of catalyst suspensions before catalytic tests was also checked with test paper strips. In all cases, aqueous suspensions appeared to be neutral.

**Table 1:** Optimization of the Catalytic Conditions

entry	deviation from standard conditions^a^	H_2_O_2_(mmol L^–1^)
1	none	2.50
2	O_2_ balloon	2.38
3	3 mL solution	0.79
4	3 mL solution + O_2_ balloon	0.44
5	3.5% methanol SED	1.40
6	no SED	0.26
7	46 μmol H_2_SO_4_	0.17
8	420 μmol AcOH	0.38
9	132 μmol HCl	0.24
10	2 mmol NaOH	0.33
11	51 μmol K_2_CO_3_	0.24

a Standard conditions: bubbled O_2_, 2 mL solution, 3.5% glycerin SED, neutral pH, 410 nm, 410 nm, r.t., 1 h.

**Figure 3:**
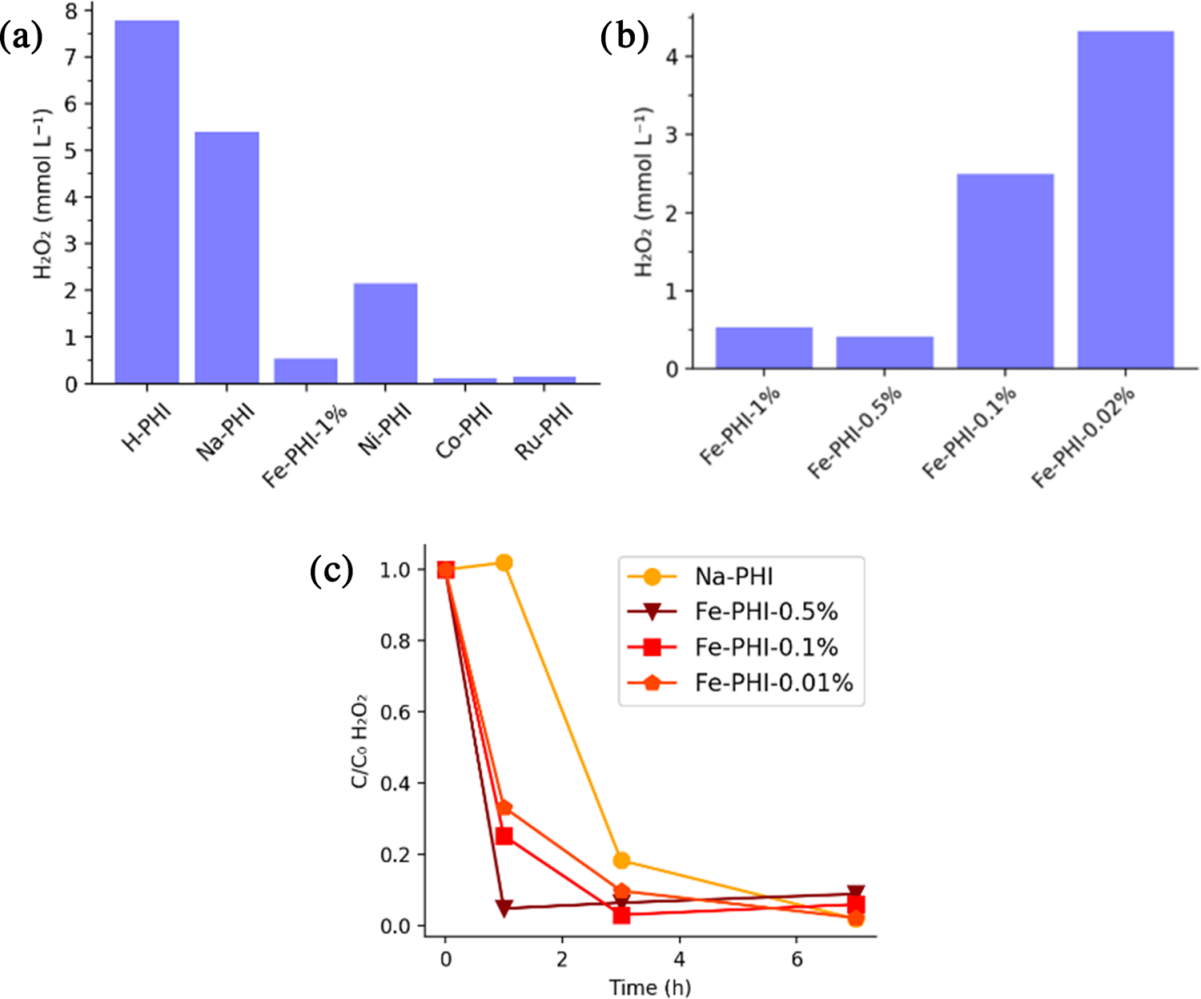
(a) Comparison of different M-PHIs in photocatalytic H_2_O_2_ production. (b) Effect of Fe-PHI metal loading on photocatalytic H_2_O_2_ production. Reaction conditions: 5 mg catalyst, 2 mL of 3.5% w/w glycerin, bubbled O_2_ 1 min before reaction, 410 nm (100 W), r.t, 1 h. (c) Effect of Fe-PHI metal loading on photocatalytic H_2_O_2_ decomposition. Reaction conditions: 5 mg catalyst, 2 mL of deionized water, 2.8 μL of 50% w/w H_2_O_2_, bubbled Ar for 3 min before reaction, 410 nm (100 W), r.t.

Among the poly(heptazine imides) investigated, only Na-PHIs, Fe-PHI-0.1%, Ni-PHIs, and H-PHIs photocatalyzed H_2_O_2_ synthesis in noticeable amounts (Figure 3a). Although the low activity of most M-PHIs is not easily rationalized, a possible explanation concerns the consecutive H_2_O_2_ decomposition to O_2_ and H_2_O. Various metals, especially silver, elements of the platinum group, and their metal oxides are known to catalytically decompose H_2_O_2_.^28,29^ Catalytic properties of iron and cobalt oxides in this reaction are well documented.^30,31^ Under the aerobic conditions of ORR, the side oxidation of single atoms on PHIs to corresponding oxides cannot be excluded. To investigate the possible side catalytic activity of these metals, the experiments were reproduced comparing Fe-PHIs with different metal loadings. Results in Figure 3b show that a lower metal content indeed enhances H_2_O_2_ yields. To further assess the role of iron in catalytic decomposition, the rate of H_2_O_2_ consumption under irradiation with Na-PHIs and Fe-PHIs was measured (Figure 3c). The amount of hydrogen peroxide was more than half after 1 h reaction in Fe-PHI suspensions and approached total conversion already after 3 h. The higher the metal loading, the faster the decomposition during the 1st hour. In contrast, no noticeable decomposition was observed with Na-PHIs after 1 h, and also subsequent conversion was slower than that in the presence of Fe-PHIs. The results suggest that the incorporation of transition metals in poly(heptazine imides) brings no improvements to the light-driven oxygen reduction to hydrogen peroxide. Possibly enhanced H_2_O_2_ production rates are overcompensated by the metal or metal oxide-catalyzed product decomposition. This can be noticed by the decrease in H_2_O_2_ production when the iron concentration increases (Figure 3b). Therefore, further experiments and mechanistic studies focused on Na-PHIs and H-PHIs.

H-PHI activity in photocatalytic H_2_O_2_ was one of the highest ever reported in the literature in terms of the production rate (Table S3). More importantly, such a high photocatalytic H_2_O_2_ production yield (1556 mmol L^–1^ h^–1^ g^–1^) was achieved with small amounts of the catalyst (5 mg). The apparent quantum yields (AQYs) measured at 410 nm for Na-PHIs and H-PHIs were, however, still only 0.45 and 0.86%, respectively. Such low values may be attributed to the very high intensity of the LED irradiation source (160 mW cm^–2^) and imperfect reaction design, e.g., the intensity-dependent excitation rate is not balanced by available oxygen molecules with their known low concentration in water. In this case, excited states would not enter the photoredox channel but relax differently with a dramatic decrease in the AQY.^32^ AQY can be potentially optimized in future tests by employing—for the sake of argument—a less powerful irradiation source or by integrating a catalyst with a three-phase boundary design, as it turned out to be very successful in similar photochemical reactions.^33^ Both Na-PHIs and H-PHIs exhibited good recyclability (Figure S5a). Interestingly, catalytic performance was enhanced after the first catalytic test (1.24- and 1.51-fold enhancement for Na-PHIs and H-PHIs, respectively). In a previous report, the photocatalytic activity of W-doped TiO_2_ nanorods was shown to increase up to 3.1 times upon catalyst recycling.^34^ A clear change of order in Na-PHIs occurred after four catalytic cycles. Na-PHIs lost their typical yellow color after two catalytic cycles and turned into a gray-white powder. The complex pattern in the 25–29° region was replaced by a single peak at 27.7° in the PXRD diffractogram of Na-PHIs recovered after four cycles. The single reflection perfectly matched that observed for H-PHIs (Figure S5b). Changes in the color and structure suggest that Na-PHIs are slowly converted into H-PHIs during the reaction due to protonation. Glycerin releases protons when oxidized to glyceraldehyde or dihydroxyacetone. In a perfectly closed cycle, those protons would combine with oxygen to yield hydrogen peroxide. However, the catalyst itself may trap the protons on anionic sites, exchanging sodium counterions with them.

### Kinetics and Preliminary Mechanistic Studies

**Figure 4:**
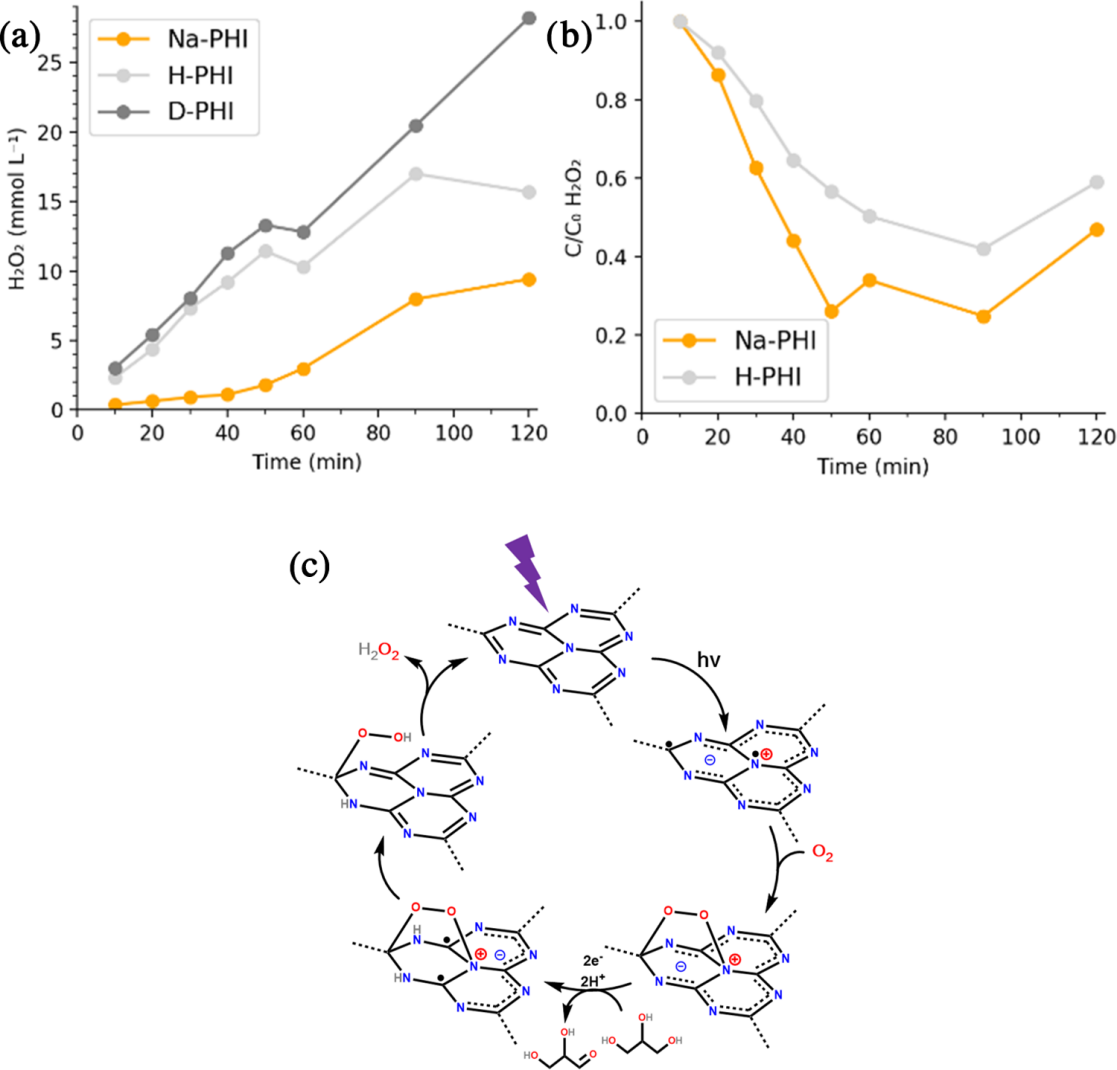
Kinetics of photocatalytic H_2_O_2_ (a) production and (b) decomposition. Reaction conditions: (a) 14 mL of 3.5% glycerin, 35 mg catalyst, bubbled O_2_ for 5 min before reaction, 410 nm (100 W), r.t; (b) 13.6 mL of 3.5% glycerin, 385 μL of 50% H_2_O_2_, 35 mg catalyst, bubbled O_2_ for 5 min before reaction, 410 nm (100 W), r.t. (c) Proposed general mechanism of photocatalysed ORR on carbon nitrides.

Photocatalytic experiments were subsequently reproduced in a single batch to properly assess the rate of H_2_O_2_ production and decomposition. Same conditions were applied except for the reaction atmosphere (O_2_ or Ar). H-PHIs showed a much faster H_2_O_2_ production than Na-PHIs (Figure 4a). Decomposition rates under irradiation in the presence of H-PHIs and Na-PHIs were very similar (Figure 4b). Therefore, it can be concluded that H-PHIs led to an intrinsic faster ORR. H_2_O_2_ synthesis seems to slow down after 120 min, possibly due to limited oxygen supply rate or concomitant H_2_O_2_ decomposition. Conversely, the concentration of H_2_O_2_ increased after 100 min of decomposition. In this case, no simultaneous ORR can occur due to the argon-purged atmosphere. Instead, some initially adsorbed H_2_O_2_ might be slowly released from the catalyst upon stirring. This observation suggests that the trapping of hydrogen peroxide within the pores of the solid catalysts should be taken into consideration. Finally, a preliminary investigation of the reaction mechanism was carried out. As a starting point, the commonly accepted scheme in the literature was considered (Figure 4c).^18,35^ Charges are separated upon irradiation, and holes are subsequently quenched by the SED (glycerin in this work). SED oxidation releases two protons, which are trapped by nitrogen atoms on carbon nitride. Molecular oxygen undergoes end-on adsorption and a swift reaction to the endoperoxo species. In the last step, electron transfer from the PHI scaffold releasing the endoperoxide species and protonation of oxygen atoms (a proton-coupled electron transfer) yield hydrogen peroxide, which is then partly released. Recently reported mechanistic studies suggested that strong adsorption of ORR intermediates may limit H_2_O_2_ light-assisted synthesis on carbon nitrides.^25^ It should be noted that H_2_O_2_ liberation is coupled to the protonation of the endoperoxide species. Therefore, the faster kinetics of H-PHIs might be explained by its higher proton content. Assuming that protons are transferred from the surface of the catalyst to the adsorbed oxygen, a kinetic isotope effect (KIE) might result from isotope replacement. To test this hypothesis, an isotope substitution experiment was designed. The kinetics experiment to test H_2_O_2_ synthesis was repeated with deuterated poly(heptazine imides) (D-PHIs) in a 3.5% glycerin solution in deuterium oxide. D-PHIs were synthesized by replacing hydrochloric acid with deuterium chloride in the H-PHI synthetic protocol. However, no substantial difference in the H_2_O_2_ production rate was observed (Figure 4a). This rules out proton transfer from protonated poly(heptazine imides) to oxygen as the rate-determining step of the reaction, while one of the two-electron transfers seems to be key. On the other hand, the oxidation of glycerin by the photogenerated hole quenching is a proton-coupled electron transfer too, and the hydrogen atoms on the catalyst in the active negatively charged state may mostly originate from glycerin.

**Figure 5:**
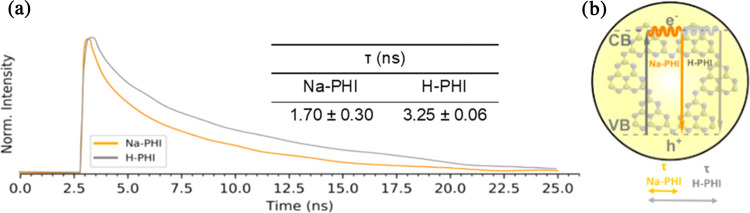
(a) Time-resolved photoluminescence decay curves of Na-PHI and H-PHI aqueous suspensions. (b) Scheme illustrating that higher τ values mean longer photoexcited carriers and enhanced electron transfer. The inset is the excited-state lifetimes of Na-PHI and H-PHI aqueous suspensions.

A further strategy to probe the mechanism involves time-resolved photoluminescence spectroscopy. Both Na-PHIs and H-PHIs exhibited fluorescence emission at 450 nm when irradiated at 405 nm. First, measurements of the excited-state lifetimes were performed on aqueous suspensions of the materials (no glycerin added). Lifetimes (τ) were intensity-averaged (see the Materials and Methods section for details). H-PHIs exhibited almost 2 times longer lifetimes than Na-PHIs (Figure 5a). Incidentally, the Na-PHI lifetime reproduced the value reported in the literature for g-C_3_N_4_ (1.71 ns, intensity-averaged).^21^ Longer excited-state lifetimes are supposed to increase the chances of electron/hole transfer to diffusing substrates before charge recombination. According to this interpretation, enhanced light-driven ORR with H-PHIs can be attributed to a suppression of charge recombination (Figure 5b). However, some authors claim that shorter lifetimes indicate a lower probability of radiative recombination pathways and thus longer-lived separated carriers.^21,36,37^ Steady-state photoluminescence measurements resulted in higher fluorescence from H-PHIs than from Na-PHIs (Figure S6). Since the two materials have very similar absorbance at the irradiation wavelength, stronger fluorescence correlates well with longer excited-state lifetimes (τ), assuming that the fluorescence decay rate constant was similar for both materials. Further discussion can be found in the Supporting Information. It can be argued that an increase of viscosity upon the addition of glycerin would promote the fluorescence lifetime. However, when glycerin was replaced with methanol at the same weight concentration, the former led to H_2_O_2_ synthesis ∼1.8 times faster than the latter (Table 1). Therefore, we believe that the glycerin concentration used in this work (3.5% w/w, 380 mmol L^–1^) is sufficiently low for viscosity effects to be relevant.

## Conclusions

PHIs, a robust and inexpensive structural version of carbon nitride, were investigated as a platform for photocatalytic two-electron oxygen reduction reaction aiming for green hydrogen peroxide synthesis. Metal-doped and fully protonated poly(heptazine imides) were synthesized via cation exchange from the same sodium poly(heptazine imide) precursor. Catalytic tests showed better performances for the precursor Na-PHI and the protonated H-PHI. This could be attributed to the tendency of transition metals to also catalyze the follow-up reaction to decompose *in situ*-generated hydrogen peroxide. This result was ultimately confirmed by tests with Fe-PHIs. The highest photocatalytic activity was achieved with H-PHIs, producing up to 1556 mmol L^–1^ h^–1^ g^–1^ H_2_O_2_ under 410 nm irradiation using glycerin as a sacrificial electron donor. This result is one of the highest reported values in the literature, which looks promising for the development of cost-effective future H_2_O_2_ applications based on carbon nitrides, especially in the energy sector. The apparent quantum yield still looks low, but it can be improved by optimized conditions, such as irradiation source intensity and reactor engineering. Secondary experiments including isotope labeling, FTIR, and Raman, as well as spectrophotochemistry indicate the key importance of two-proton-coupled electron transfers from the catalyst to an endoperoxide species, which explain the high selectivity of the reaction, very similar to the related anthraquinone process, while H-PHIs can serve as a local proton donor at the same time. This makes the hardly examined H-PHI catalyst an interesting case for all reactions where photoredox chemistry and proton transfer have to be optimized at the same time.

## Materials and Methods

### Synthesis of Sodium Poly(Heptazine Imides) (Na-PHIs)

All of the chemicals were purchased from Sigma-Aldrich. Melamine (21 g) was thoroughly ground with NaCl (210 g) in a mortar with a pestle and then additionally shaken by ball milling at a frequency of 25 s^–1^. The powder was transferred into a porcelain crucible, which was covered with a lid. The crucible was placed in an oven and heated under a constant nitrogen flow (1 L min^–1^) to 600 °C with a heating rate of 2.3 °C min^–1^, held at 600 °C for 4 h, and then allowed to cool down at a rate of 10 °C min^–1^. The crude yellow product was removed from the crucible and stirred in deionized water for 3 h at 95 °C. The suspension was then centrifuged at 9000 rpm for 5 min, washed with 1 L of deionized water, and dried in vacuum at 60 °C overnight.^15,38^

### Synthesis of Metal-Doped Poly(Heptazine Imides) and Protonated Poly(Heptazine Imides) via Cation Exchange

Fe-PHIs, Ni-PHIs, Co-PHIs, and Ru-PHIs were prepared by mixing previously synthesized Na-PHIs with aqueous solutions of FeCl_3_·6 H_2_O, NiCl_2_, CoCl_2_, and RuCl_3_, respectively. For each material, 200 mg of Na-PHIs was transferred to a 15 mL centrifuge tube, mixed with 4 mL of metal salt solution, and immediately shaken vigorously to homogenize the suspension. A concentration of 10 mM of salt was used for Fe-PHI-1%, Ni-PHI-1%, Co-PHI-1%, and Ru-PHI-1%. FeCl_3_·6 H_2_O solutions (5, 1, and 0.1 mM) were used to synthesize Fe-PHI-0.5%, Fe-PHI-0.1%, and Fe-PHI-0.02%, respectively. H-PHIs were synthesized by replacing the 4 mL metal salt solution by a 2 mL of 37% aqueous hydrochloric acid. D-PHIs were synthesized by replacing hydrochloric acid with 37% deuterium chloride in deuterium oxide. The suspensions were sonicated for 1 h and centrifuged at 9000 rpm for 5 min, washed with about 5 mL of deionized water, and centrifuged again twice. Finally, they were dried in vacuum at 60 °C overnight.^19,23,39^

### Characterization

The powder X-ray diffraction (PXRD) patterns were recorded on Bruker D8 Advance diffractometer equipped with a scintillation counter detector with Cu Kα radiation (λ = 0.15418 nm) applying a 2θ step size of 0.05° and a counting time of 3 s per step. Steady-state UV–vis absorption spectra were acquired using Shimadzu UV 2600 in diffuse reflectance mode. FTIR spectra were recorded on a Nicolet Summit FTIR spectrometer equipped with a diamond attenuated total reflection unit. Inductively coupled plasma-optical emission spectrometry (ICP-OES) was conducted using a Horiba Ultra 2 instrument equipped with photomultiplier tube detector. Samples were dissolved in aqua regia before analysis. X-ray photoelectron spectroscopy (XPS) measurements were performed on a Thermo Scientific Escalab 250 Xi. A microfocused, monochromated Al Kα X-ray source (1486.68 eV) and a 400 μm spot size were used in the analysis. Samples were prepared using carbon tape. LiCl was added to each sample to calibrate the binding energies toward Li. Thermo Scientific Advantage software was used to analyze the resulting spectra. The Mott–Schottky measurements were performed in a Biologic MPG-2 system using a three-electrode setup consisting of a Pt wire working as the counter electrode, an Ag/AgCl as the reference electrode, and a F-doped tin oxide (FTO) glass coated with the material as the working electrode. The working electrode was prepared on FTO glass that was cleaned by sonication in ethanol for 30 min and dried at 353 K. The boundary of FTO glass was protected using Scotch tape. The 3 mg sample was dispersed in 0.2 mL of water by sonication to get a slurry mixture with 20 μL of Nafion. The slurry was spread onto pretreated FTO glass. After air-drying, the Scotch tape was removed and the working electrode was further dried at 393 K for 2 h to improve adhesion. Raman spectra were recorded using a confocal Raman microscope alpha300 (WITec, Germany) coupled with laser excitation at 785 nm. The laser beam was focused through a Zeiss EC Epiplan-Neofluar 10x microscope objective lens. The Raman spectra have been measured with an integration time of 1 s under an excitation laser power of 42 mW. The spectra were acquired with a thermoelectrically cooled DR316B-LD, DD CCD detector placed behind the spectrometer UHTS 300S from WITec, using a grating with a resolution of 600 grooves/mm. For high-resolution transmission electron microscopy (TEM) observations, a suspension of the samples in ethanol was sonicated for 10 min and then drop-cast to a Cu grid with a lacey carbon support and dried for 5 min. The TEM study was performed using a double Cs corrected JEOL JEM-ARM200F (S)TEM operated at 80 kV and equipped with a cold-field emission gun. Time-resolved fluorescence measurements were performed using a single-photon counting setup (TCSPC) with a Becker–Hickl PML-spectrometer (modified Oriel MS-125) with a laser repetition rate of 2 MHz. The sample (2 mg) was dispersed in 2 mL of deionized water in a capped cuvette. The detector comprises a Becker–Hickl PML-16-C-1 (modified Hamamatsu) multi-alkaline photomultiplier. The excitation wavelength was 405 nm. The excitation was carried out using a pulsed laser diode at 30 nJ cm^–2^ (LDH-P-C405, PicoQuant GmbH). The emission was recorded at 450 nm. Raw decay data presented as the logarithm of photon counts versus time were analyzed with data analysis software of PicoQuant GmbH (Germany). The decay times were extracted by means of a deconvolution fit based on a double exponential model.^21^ Considering that
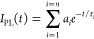
where *a*_*i*_ and *τ*_*i*_ are the amplitude and the lifetime of the *i*th component, respectively, the intensity-averaged fluorescence lifetime τ was calculated as
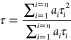


### Photocatalytic H_2_O_2_ Production

In a typical test, 5 mg of catalyst was dispersed in 2 mL of a 3.5% w/w glycerin aqueous solution in a 4 mL vial. Oxygen was bubbled in the solution for 1 min through a septum using a needle. The solution was then irradiated under stirring at 410 nm using two LED lamps (50 W each) for 1 h. The suspension was then centrifuged at 10 000 rpm for 10 min in a 2 mL Eppendorf tube, and H_2_O_2_ in the supernatant was quantified spectrophotometrically, as described below.

### Apparent Quantum Yield (AQY) Determination

Experiments for the determination of the quantum yield were performed under the same conditions described above. The 4 mL glass vial was put all the way down in a slot of a black vial holder and illuminated from the bottom with a 410 nm LED. The intensity of the irradiation source (160 mW cm^–2^) was measured on a Thorlabs PM400 power meter console. The glass vial had a cylindrical shape with a diameter of 1.52 cm.

### H_2_O_2_ Decomposition on Na-PHIs and Fe-PHIs

In a typical test, 5 mg of catalyst was dispersed in a ≈50 mM H_2_O_2_ solution in a 4 mL vial. Argon was bubbled in the solution for 3 min through a septum using a needle. The vial was then irradiated at 410 nm using an LED lamp (160 mW cm^–2^). After a given time, the suspension was then centrifuged at 10 000 rpm for 10 min in a 2 mL Eppendorf tube and H_2_O_2_ in the supernatant was quantified spectrophotometrically, as described below.

### H_2_O_2_ Colorimetric Determination

Hydrogen peroxide was quantified spectrophotometrically following the titanium oxalate method. A 10 g L^–1^ solution of titanium oxide oxalate dihydrate (K_2_[TiO(C_2_O_4_)_2_]·2H_2_O) in diluted sulfuric acid was prepared according to a reported protocol.^40^ This reagent (3 mL) was transferred to a 4 mL vial. The supernatant (1 mL) from previously centrifuged suspension was added to the reagent. The resulting solutions, properly diluted when needed, were analyzed using the UV–vis spectrometer, collecting absorbance values at 400 nm. A calibration curve was made with external samples of known concentrations. The analytical response was linear in the range of concentrations (i.e., 0.7–4.4 mmol L^–1^).

### Recyclability Tests

Each catalytic cycle was performed under the typical conditions described above. After each cycle, the catalyst was dried in vacuum at 60 °C overnight. To add the same mass of catalyst at each cycle, a decreasing number of replicas for each cycle was possible due to catalyst loss. Error bars were obtained from 4, 3, and 2 replicas at 1st, 2nd, and 3rd cycles, respectively.

### Kinetics and Isotope Substitution Experiment

In a typical test, 35 mg of catalyst was transferred in a 25 mL round-bottom flask, followed by the addition of 14 mL of 3.5% glycerin to test H_2_O_2_ synthesis or 13.6 mL of 3.5% glycerin and 385 μL of 50% H_2_O_2_ to test decomposition. The flask was capped with a septum. and gas was bubbled through it with a needle for about 5 min. Oxygen was bubbled to test H_2_O_2_ synthesis, while argon to test decomposition. Hence, the needle was removed and the suspension was stirred under irradiation at 410 nm with two 50 W LED lamps. For every point of the kinetics curve, a sample of about 1.5 mL was transferred with a syringe from the flask to a 2 mL Eppendorf tube and H_2_O_2_ in the supernatant was quantified spectrophotometrically. The isotope substitution experiment to probe KIE in H_2_O_2_ production was performed by replacing deionized water with deuterium oxide and H-PHIs with D-PHIs.

## Supporting Information Available

Scheme of synthetic protocol and chemical structures of Na-PHI and H-PHI; metal loading of the synthesized M-PHIs; bandgaps and band edges of the prepared poly(heptazine imides); TEM images of Na-PHI and H-PHI; pictures of Na-PHI and H-PHI powders; Tauc and Mott–Schottky plots of Na-PHI and H-PHI; XPS spectra of Na-PHI and H-PHI; comparison of results from this work with the state of the art in photocatalytic H_2_O_2_ synthesis; Na-PHI and H-PHI recyclability tests; comparison of PXRD diffractograms of Na-PHI before reaction and after 4 catalytic cycles; and steady-state photoluminescence of Na-PHI and H-PHI aqueous suspensions (PDF)
