# Supplementary material for: Modified Poly(Heptazine Imides): Minimizing H2O2 Decomposition to Maximize Oxygen Reduction
Source: ACS Appl Mater Interfaces. 2022 Oct 31;14(44):49820–9. doi: 10.1021/acsami.2c14872 (PMC9650642; doi:10.1021/acsami.2c14872)
Supplement: Supplementary file 1 — am2c14872_si_001.pdf [file am2c14872_si_001.pdf]

Supporting Information

Modified poly (heptazine imides): Minimizing H_2O_2 decomposition to maximize oxygen reduction

Andrea Rogolino[a], Ingrid F. Silva[b], Nadezda V. Tarakina[b], Marcos A. R. da Silva,[c] Guilherme F. S. R. Rocha,[c] Markus Antonietti[b], Ivo F. Teixeira[c,*]

[a]*Galilean School of Higher Education, University of Padova, Via Venezia 20, Padova, 35131, Italy*

[b]*Department of Colloid Chemistry, Max Planck Institute of Colloids and Interfaces, Am Mühlenberg1, Potsdam, 14476, Germany*

[c]*Department of Chemistry, Federal University of São Carlos, Washington Luis Highway s/n Km 235, SãoCarlos, 13565-905, São Paulo, Brazil*

*Corresponding author
Email address: ivo@ufscar.br (Ivo F. Teixeira)

S.1. Na-PHI and H-PHI structures

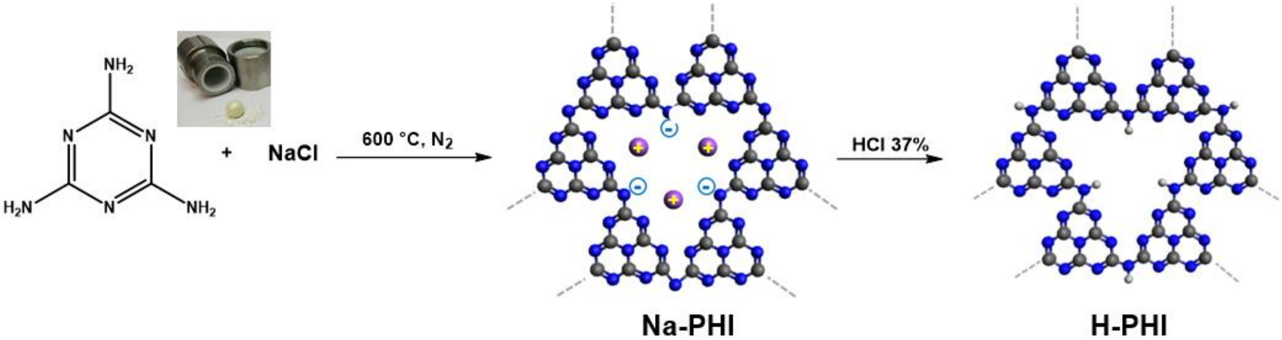

Figure S.1: Scheme of synthetic protocol and chemical structures of Na-PHI and H-PHI. Please note that dotted lines in chemical structures indicate a periodic lattice.

S.2. Metal loading, band gaps and band positions of M-PHIs

Table S.1: Metal loading of the synthesized M-PHIs

Material	%Na	%M
Na-PHI	9.7	/
Fe-PHI-1%	6.02	0.94
Fe-PHI-0.5%	6.34	0.50
Fe-PHI-0.1%	6.75	0.13
Fe-PHI-0.02%	6.60	0.02
Ni-PHI	4.36	1.08
Co-PHI	5.77	0.88
Ru-PHI	3.87	1.52

Table S.2: Band gaps and band edges of the prepared poly (heptazine imides)

Material	Band gap (eV)	Conduction band (E vs NHE)	Valence band (E vs NHE)
Na-PHI	2.78	-0.24	2.54
H-PHI	3.00	-0.19	2.82
Fe-PHI	2.82	-0.11	2.71
Ni-PHI	2.82	-0.93	1.90
Co-PHI	2.78	-1.25	1.53
Ru-PHI	2.87	-0.61	2.26

S.3. TEM images of Na-PHI and H-PHI

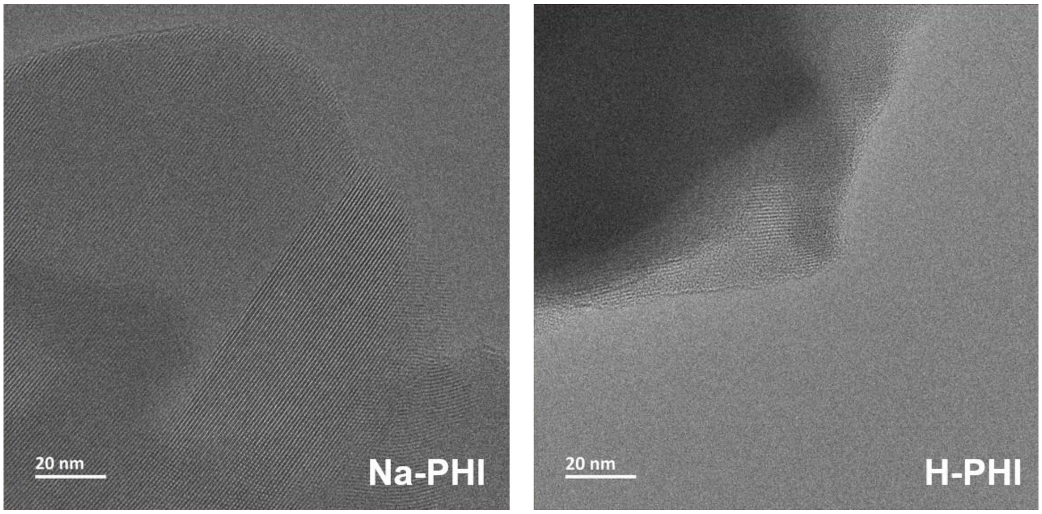

Figure S.2: Transmission electron microscopy (TEM) images of Na-PHI and H-PHI.

S.4. Pictures, Tauc and Mott-Schottky plots of Na-PHI and H-PHI

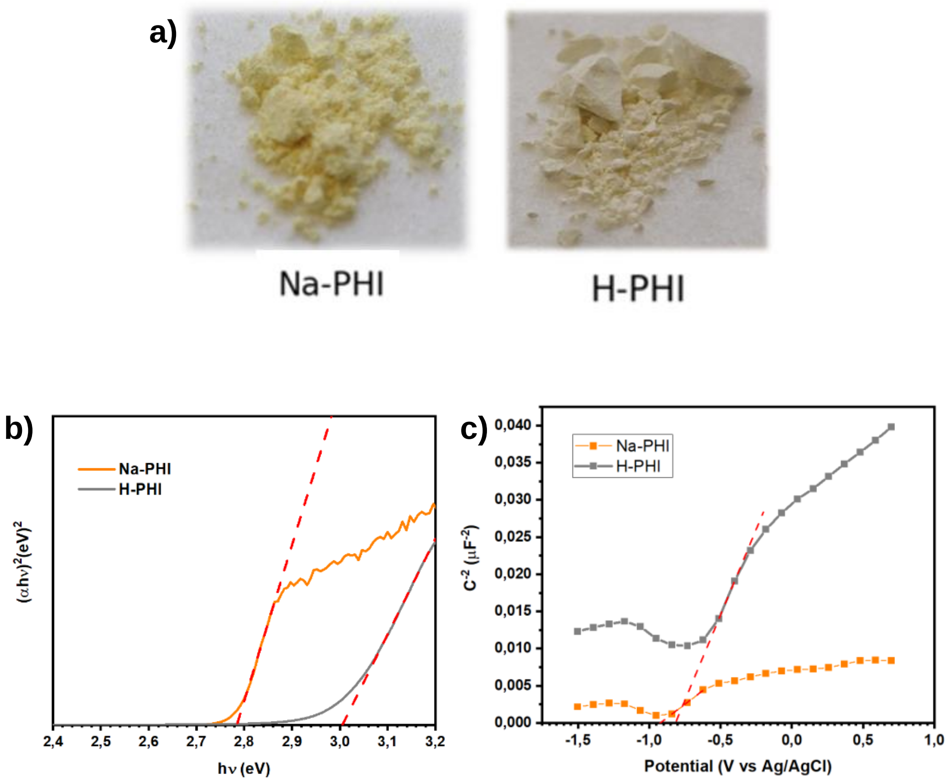

Figure S.3: Pictures of Na-PHI and H-PHI powders. B) Tauc and c) Mott-Schottky plots of Na-PHI and H-PHI.

S.5. XPS spectra of Na-PHI and H-PHI

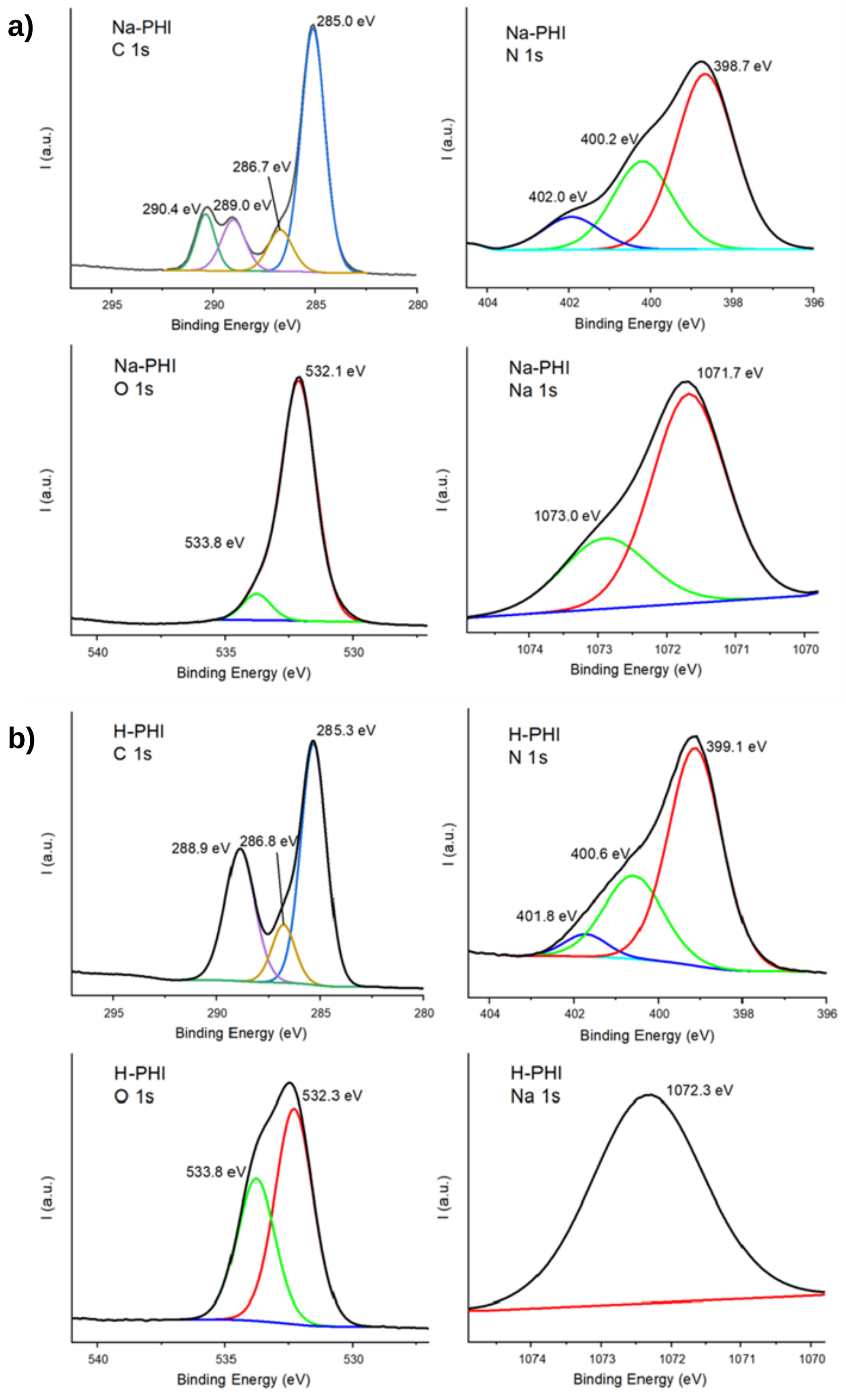

Figure S.4: XPS spectra of a) Na-PHI and b) H-PHI.

C1s signal in Na-PHI resulted from four distinguished contributions at 285.0, 286.7, 289.0and 290.4 eV, arising from adventitious carbon, surface C-OH groups, CN_3 carbons in the aromatic rings and likely surface carbonate groups O=C-O deriving from excessive oxygenation in the synthetic procedure.[1] Interestingly, the latter peak disappeared in H-PHI, possibly due to acid-catalysed decarboxylation after the addition of hydrochloric acid in the synthetic procedure. Both Na-PHI and H-PHI showed three signals at around 398.9, 400.4 and 401.9 eV in the N1s spectrum, ascribed to NC_2 nitrogens in the aromatic rings, sp^3 nitrogens in NC_3 groups and amino groups -NH and $-NH_2$. Hydroxyl groups and adsorbed water were assigned to the peaks at 532.2 and 533.8 eV respectively in the O1s signal. Unfortunately, contributions from anionic C^-, N^- and O^-, which may have marked a clear distinction between Na-PHI and H-PHI, could not be observed in C1s, N1s and O1s spectra. Indeed, such peaks were observed only at very low intensities in poly (heptazine imides).[2] Finally, two distinct contributions were detected in the Na1s signal of Na-PHI, while only one broad gaussian appeared for H-PHI. This might indicate the location of sodium cations in two different environments, tentatively assigned to randomly distributed or interlayer and nitrogen-coordinated Na^+, in Na-PHI. Signal from sodium in H-PHI would then derive from residual uncoordinated Na^+.

S.6. Comparison with the state of the art

Table S.3: Comparison of results from this work with the state of the art in photocatalytic H_2O_2 synthesis

Entry	Catalyst	Irradiation source	Sacrificial electron donor	H_2O_2 (µM h^{-1}) (mass of catalyst)	% AQY	Ref.
1	TiO_2	> 280 nm	Benzyl alcohol	3350 (50 mg)	29.1@334 nm	48
2	TiO_2/rGO	> 320 nm	2-Propanol	1320 (20 mg)	/	62
3	$Cd_3(C_3N_3S_3)_2$	> 420 nm	Methanol	2188 (80 mg)	/	57
4	g-C_3N_4	> 420 nm	Ethanol	500 (20 mg)	12@420nm	58
5	g-C_3N_4 with N vacancies	> 400 nm	Ethanol	1150 (200 mg)	/	61
6	OCN	> 420 nm	2-Propanol	1200 (50 mg)	10.2@420nm	59
7	g-C_3N_4 /PDI/rGO-BN	> 420 nm	2-Propanol	3095 (50 mg)	/	63
8	PCN-NaCN	Solar simulator	Glycerin	187 (10 mg)	11.8@420nm	64
9	ACNN	> 420 nm	2-Propanol	5100 (25 mg)	30.7@429nm	65
10	g-C_3N_4 -PWO	> 420 nm	/	1007 (100 mg)	/	66
11	g-C_3N_4 /PDI/rGO-BN	> 420 nm	/	500 (250 mg)	7.3@420nm	63
12	Sb-SAC	> 420 nm	/	182 (100 mg)	17.6@420nm	67
13	Na-PHI	410 nm	Glycerin	5386 (5 mg)	0.45@410nm	This work
14	H-PHI	410 nm	Glycerin	7779 (5 mg)	0.86@420nm	This work

S.7. Recyclability

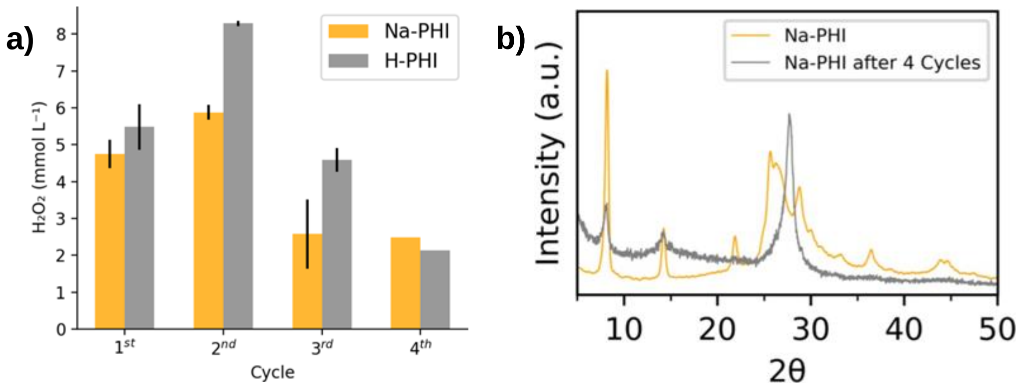

Figure S.5: a) Na-PHI and H-PHI recyclability tests. b) Comparison of PXRD diffractograms of Na-PHI before reaction and after 4 catalytic cycles.

S.8. Photoluminescence spectroscopy

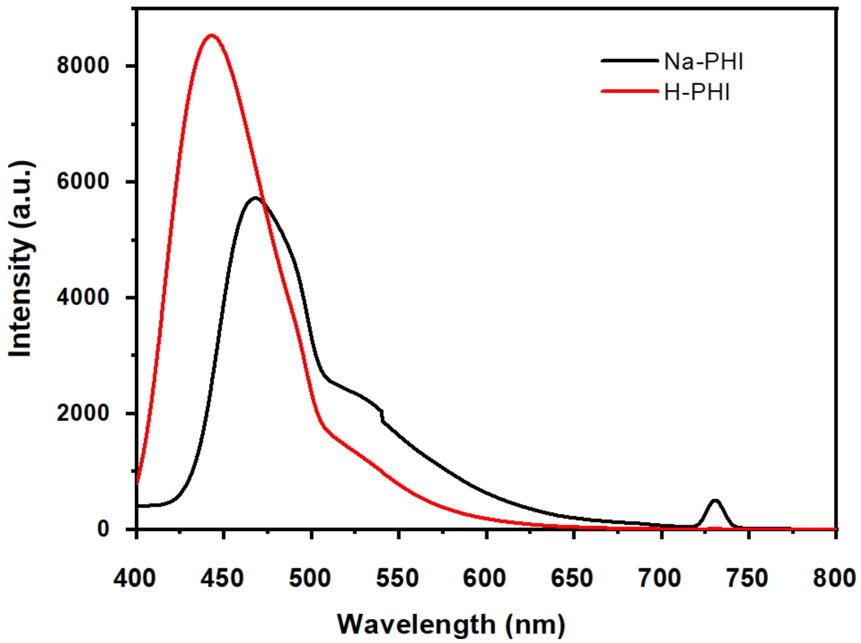

Figure S.6: Steady-state photoluminescence of Na-PHI and H-PHI aqueous suspensions

Fluorescence intensity at a given wavelength is proportional to the product of absorbed intensity and fluorescence yield (Φ_F):

$$I_F(\lambda) \propto I_{Abs} \cdot \Phi_F$$

Where Φ_F is defined as:

$$\Phi_F = \frac{\tau}{\tau_F}$$

Where τ is the excited state lifetime and τ_F is the intrinsic fluorescence lifetime, or the inverse of the fluorescence decay rate constant (k_F).

I_{abs} can be assumed to be the same for both Na-PHI and H-PHI at the irradiation wavelength (405 nm). Therefore, a higher fluorescence intensity correlates with higher fluorescence yield. Assuming τ_F is similar for the two materials, the higher fluorescence yield of H-PHI can be explained by a higher tendency of the material to preserve its excited state, possibly through additional states induced by defects in its structure. The longer the excited state lifetime, the higher the electron transfer probability.

As far as the quantum yield is concerned, the theory of excited state can help understanding the low values obtained in this study. Given n_{S1} and n_{S0} the population of excited and ground state, respectively, the following kinetic law holds:

$$\frac{dn_{S1}}{dt} = k_A n_{S0} - (k_F + k_{nr}) n_{S1}$$

Where k_A is the absorption rate constant, k_F is the fluorescence decay rate constant (= $1/\tau_F$) and k_{nr} is

the non-radiative decay rate constant (which comprises decay rate constants other than fluorescence, including phosphorescence and electron transfer). k_A is proportional to the irradiation intensity, while k_F can be split into two contributions, namely intensity-independent spontaneous emission and intensity-proportional stimulated emission:

$$k_A = B \cdot \rho(\lambda)$$

$$k_F = A + B \cdot \rho(\lambda)$$

Where A and B are constants and ρ(λ) is the irradiation intensity.

Under continuous illumination a steady-state is reached, where the population of the excited state does not vary with time. Hence:

$$k_A n_{S0} = (k_F + k_{nr}) n_{S1} \ \Rightarrow \ n_{S1} = \frac{k_A}{k_F + k_{nr}} n_{S0} \ \Rightarrow \ n_{S1} = \frac{B \cdot \rho(\lambda)}{A + B \cdot \rho(\lambda) + k_{nr}} n_{S0}$$

For high intensity irradiation sources (ρ(λ) >> 0):

$$n_{S1} \approx \frac{B \cdot \rho(\lambda)}{B \cdot \rho(\lambda)} n_{S0} \ \Rightarrow \ n_{S1} \approx n_{S0}$$

That is, the population of excited state can be at most equal to the population of the ground state. Beyond this threshold, no more electrons can be excited from the ground state to the excited state, regardless of the irradiation intensity.

The calculation of the apparent quantum yield (AQY) is made under the assumption that all absorbed photons generates excited states when absorbed. However, this assumption is not valid under very high irradiation intensity. It can be concluded that the low AQYs obtained in this work might derive from excessive source intensity. Better results will be found after optimizing the conditions, as suggested in the main text.
The decay curves (Figure 5a) were fitted using a nonlinear method with a multicomponent decay law

given by

I (t) = a1 exp(-t/τ1) + a2 exp(-t/τ2) + a1 exp(-t/τ3)

References

[1] Payne, R.; Beamson, G.; Parallel Electron Energy-loss Spectroscopy and X-ray Photoelectron Spectroscopy of Poly(ether ether ketone), *Polymer* **34** (1993) 1637–1643. doi:https://doi.org/10.1016/0032-3861(93)90321-Z.

[2] Chen, Z.; Savateev, A.; Pronkin, S.; Papaefthimiou, V.; Wolff, C.; Willinger, M.; Willinger, E.; Neher, D.; Antonietti, M.; Dontsova, D. “The Easier the Better”:

Preparation of Efficient Photocatalysts - Metastable Poly(heptazine imide) Salts, *Advanced Materials* **29** (2017) 1700555. doi:https://doi.org/10.1002/adma.201700555.
